# Novel calculation methods for geometrically accurate thread depth

**DOI:** 10.1038/s41598-026-53095-1

**Published:** 2026-05-18

**Authors:** Mátyás Andó

**Affiliations:** Institute of Computer Science, Faculty of Informatics, ELTE Eötvös Loránd University, Budapest, Hungary

**Keywords:** CNC machining, Thread depth calculation, Thread tolerances, External and internal threads, Good at first, Green manufacturing, Engineering, Materials science

## Abstract

In advanced manufacturing systems, particularly High-Mix Low-Volume (HMLV) environments, achieving first-pass conformity in threaded components remains challenging due to strict ISO tolerance requirements, non-standardized tool geometries, and reliance on gauge-based iterative corrections. These limitations not only reduce machining accuracy but also increase setup time, energy consumption, and material waste. This study introduces three fundamentally new calculation methods that enable precise geometrical determination of thread depth for CNC turning and milling of both external and internal metric threads. The methods integrate ISO-defined pitch-diameter tolerances, detailed tool geometry, and measured nose radius into a unified analytical framework, thereby enabling automatic and dimensionally accurate G-code generation. Method 1 addresses external thread turning using full-profile inserts. Method 2 provides a generalized model for external thread milling with free-form radii. Method 3 extends the approach to internal threads by incorporating pre-machined reference diameters. Industrial case studies demonstrates that the proposed methods can enhance precision manufacturing, eliminate trial-and-error adjustments. The results contribute to advanced and green manufacturing practices by improving automation readiness, shortening machine setup cycles, and enabling reliable production of atypical thread sizes and tolerance classes.

**Supplementary Information:**

The online version contains supplementary material available at 10.1038/s41598-026-53095-1.

## Introduction

Considering recent societal transformations, an increasing number of manufacturers must adapt to High-Mix Low-Volume (HMLV) production environments^1,2^. Fulfilling orders consisting of only a few pieces (often between one and ten pieces) has become a routine task for many small enterprises. A significant portion of manufacturing activities is still performed using CNC machining, and G-code programming has an increasing level of digital support and automation^3–6^.

Due to the increasing product requirements and the stricter quality inspection, manufacturing companies must achieve higher levels of precision in production. At the same time, the continuous pressure of market competition demands greater efficiency in manufacturing processes. Within this industrial environment, a critical research question arises: can threaded connections be produced rapidly and with high accuracy?

The process of threading currently involves verifying the finished thread using threaded gauges. For example, according to ISO 1502, the GO screw plug gauges shall enter the whole length (without excessive force) and NO-GO screw plug gauge must not enter more than two turns^7^. If the product does not meet the required specifications, the tool parameters or the G-code are adjusted, and a new part is machined. This trial-and-error procedure is repeated until the thread falls within the acceptable tolerance range, which is time-consuming. Moreover, the first few parts often do not meet the requirements and therefore become waste. This not only raises production costs but also implies that the energy invested in producing the raw material and its primary form has been wasted.

The processes of thread turning^8–11^ and thread milling^12–14^ are under continuous research. However, most studies primarily focus on the cutting conditions rather than on the conformance to tolerances. Although thread milling or turning research occasionally addresses thread depth^15,16^, these investigations generally do not extend to the tolerance. Recent measurement techniques evaluate thread shape^17,18^, or pitch diameter^19^ but do not provide information about the exact thread depth.

In case of the modern CNC machining processes, tool setter (traditional, or laser based) or external tool setter (or presetter) is used^20–22^. These tool setters can primarily measure tool overhanging and diameter. Moreover, external CCD camera-based tool setters (e.g. Zoller, Haas, Haimer, Kelch) can measure the insert radius with an accuracy of up to 10 micrometres.

Based on the literature, existing methods are insufficient to provide the necessary tolerance based thread depth during G-code generation. In this research, new calculation methods were developed, to provide geometrically ideal thread depth for CNC turning and milling. These models take into account the tool geometry and thread tolerances. To produce perfect parts on the first-time, an exact tool path is required, along with an accurate tool setter, a precise CNC machine and a well-skilled workers.

## Thread shape and tolerance zone

**Fig. 1:**
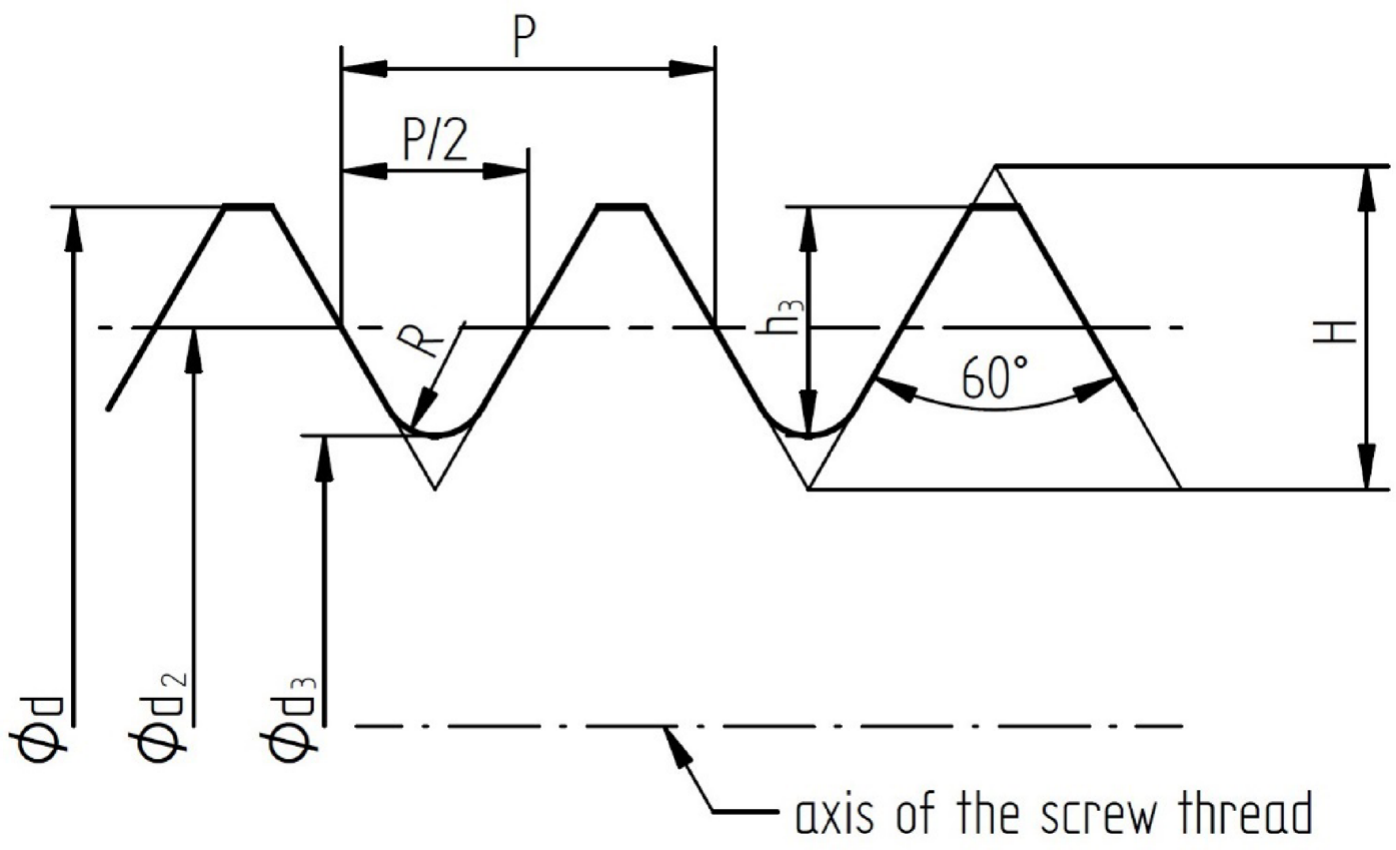
Standard ISO metric external thread – main dimensions.

For external thread with fully rounded root, the ISO 68 − 1:2023 standard^23^ provides exact information about the profile shape (Fig. 1 – the original figure from the standard was modified to present the fundamental triangle height). The thread height (h_3_) can be calculate using Eq. (1), and the root radius using Eq. (2). The standard also defines the minimum root radius for partially rounded root, with Eq. (3).

1 $$\:{h}_{3}=\frac{17}{24}\cdot\:H=\mathrm{0,613434661}\cdot\:P$$

2 $$\:R=\frac{1}{6}\cdot\:H=\mathrm{0,14434}\cdot\:P$$

3 $$\:{R}_{min}=\frac{1}{7}\cdot\:H\approx\:\mathrm{0,125}\cdot\:P$$

where P is the pitch, H is the fundamental triangle height, ød is the nominal diameter, ød_2_ is the pitch diameter, ød_3_ is the minor diameter according to ISO 5408:2009^24^.

**Fig. 2:**
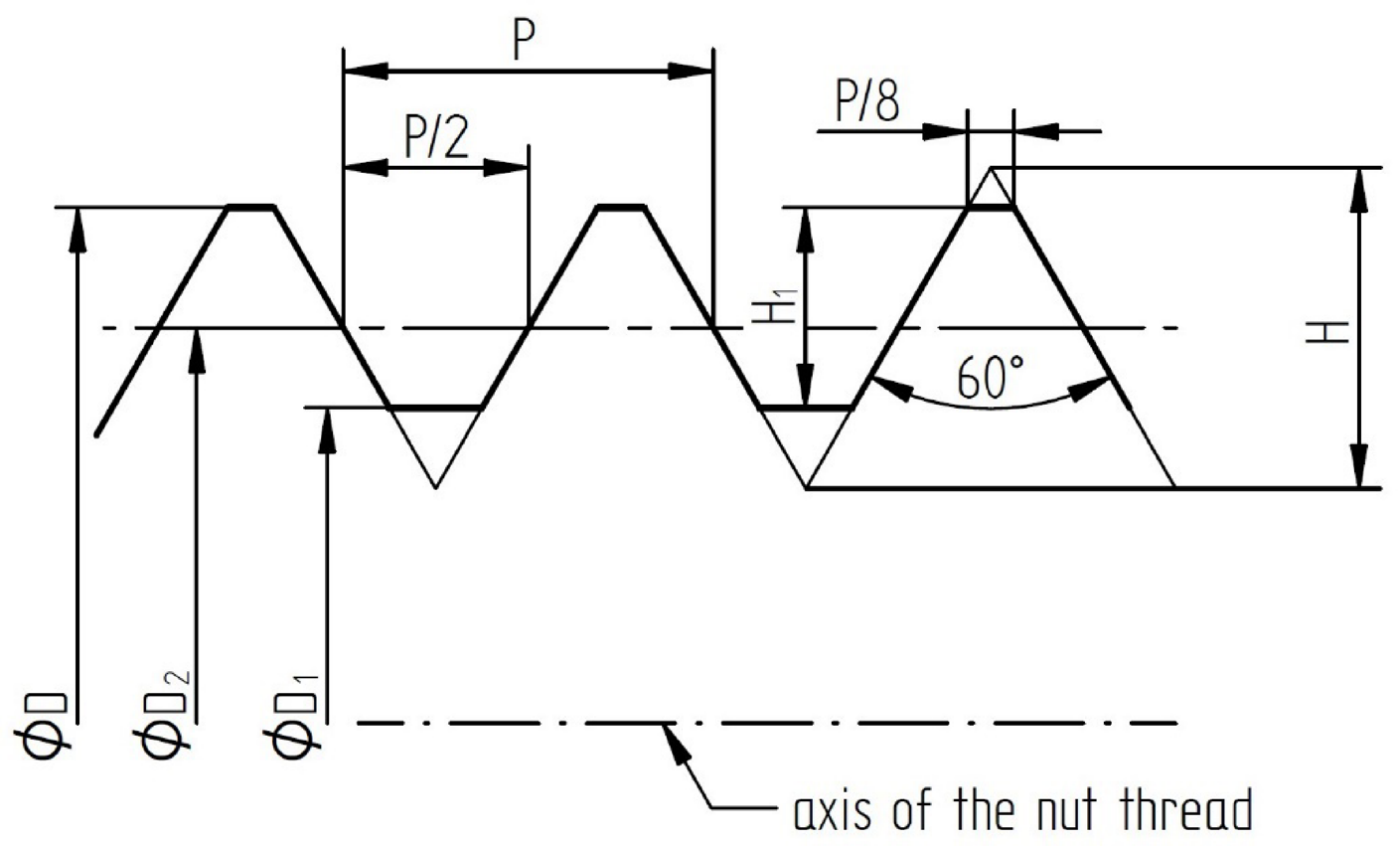
Standard ISO metric internal thread – main dimensions.

On the other hand, in case of the internal threads, the ISO 68 − 1:2023 standard provides greater flexibility regarding crest and root truncation. In this case there are no radius requirements (Fig. 2– the original figure from the standard was modified to present the fundamental triangle height). The height of the thread (H_1_) can be calculated using Eq. (4).

4 $$\:{H}_{1}=\frac{5}{8}\cdot\:H$$

**Fig. 3:**
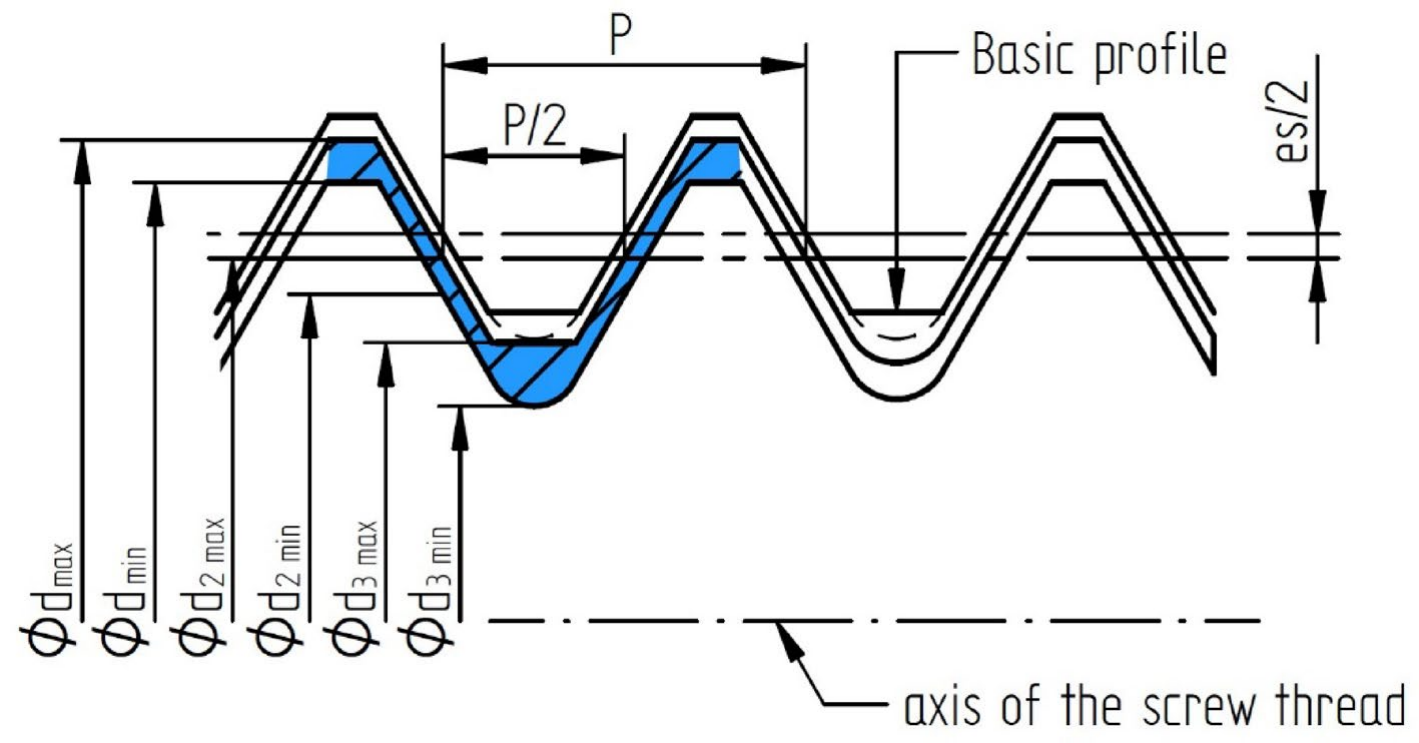
External thread tolerances in case of e, f, g.

øD is the nominal diameter, øD_2_ is the pitch diameter, øD_1_ minor diameter. The thread tolerances are defined by ISO 965-1:2026 standard. Figure 3 shows an external thread with a fundamental deviation (es), typically specified as 6 g, but it can be also *e* or *f*. The original figure from the standard was modified (diameter, pitch, and basic profile) to improve the illustration of the tolerance zone position, which is indicated in blue. It can be clearly seen that the minor diameter exhibits the largest possible variation.

**Fig. 4:**
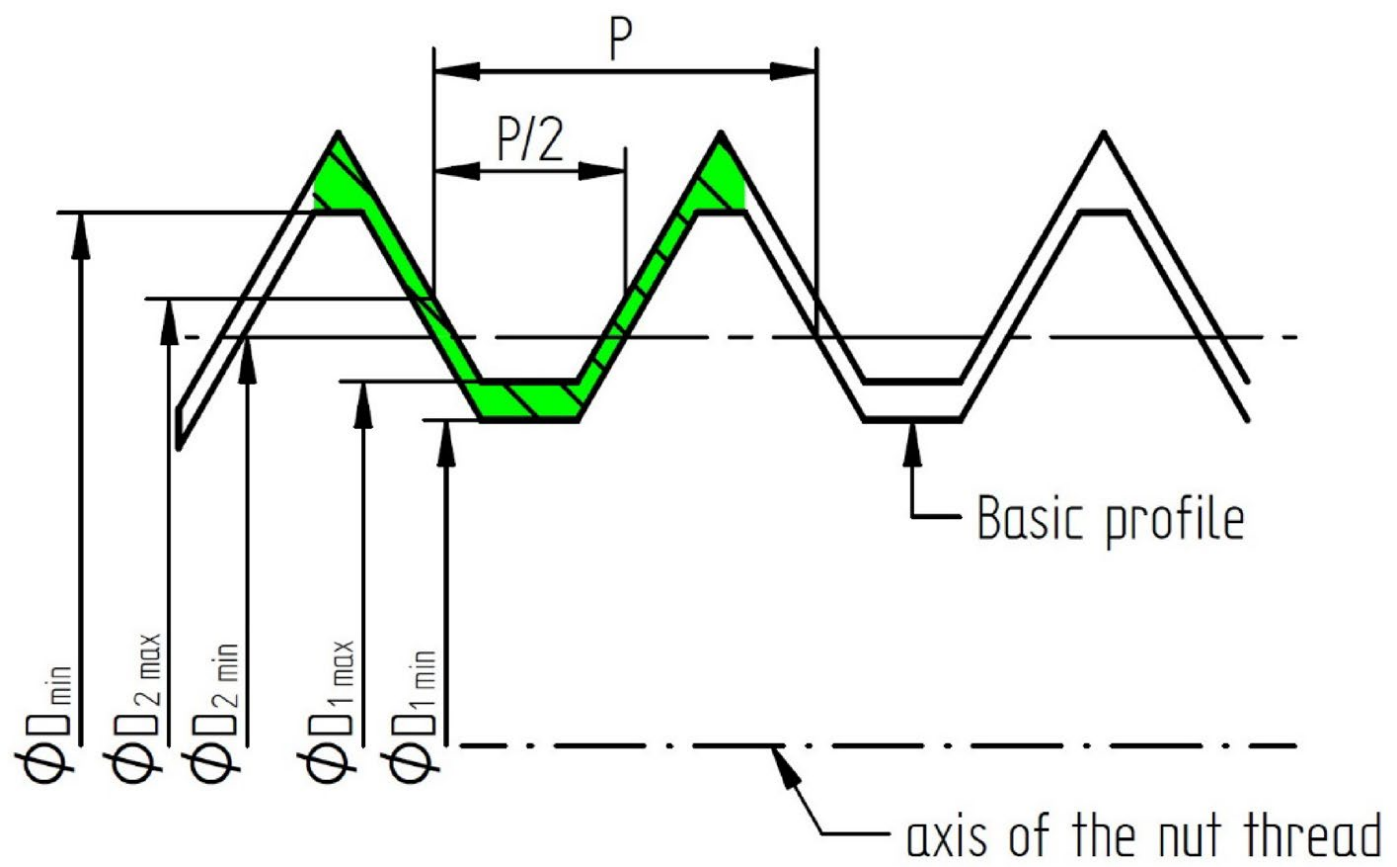
Internal thread tolerances for tolerance class H.

Figure 4, illustrates an internal thread (with the same modification like Fig. 3), where the fundamental deviation is not applied, because 6 H used as the default tolerance class. In this case, the major (nominal) diameter has only a minimum limit, while the maximum limit is determined by the thread profile itself.

According to the standard, the pitch diameters (ød2 and øD2) have the tightest tolerance limits, while the major and minor diameters are allowed greater deviations (Figs. 3 and 4). This is critical, because during the production these pitch diameters must be controlled with the highest accuracy. If the tool produces a standard thread shape, and the pitch diameter is within tolerance, then the entire thread will be within specification related to the dimensional requirements. To achieve this condition, the G-code should include the geometrically accurate total depth of cut (ap), which precisely guides the cutting insert to the middle of the pitch diameter tolerance zone. This is the core idea of the new method, based on the ideal geometrical shapes.

Although all threads have tolerances, cutting tool catalogues do not provide this information or link it with the appropriate total depth of cut. Most tool manufacturers offer only rough recommendations without any explanation or calculation. This creates an error in the G-code, therefore this inaccuracy appears on the workpiece as well. Based on the new method, this problem can be eliminated, enabling the creation of dimensionally perfect, well-defined G-code. However, additional practical problems still occur during threading, so this does not automatically guarantee a perfect thread quality.

## Method 1 for turning external threads

**Fig. 5:**
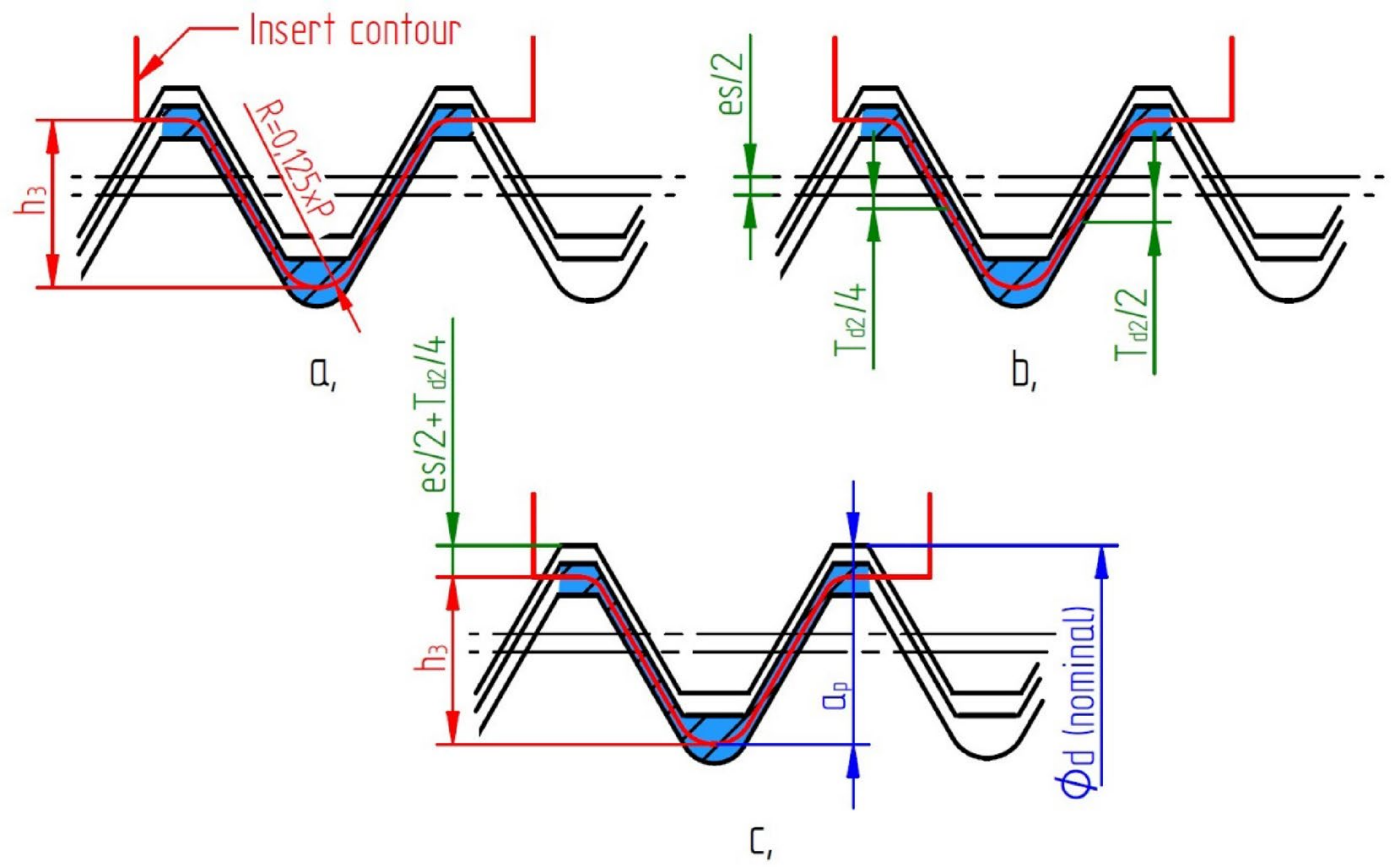
Optimal position of the final cut: (**a**), insert geometry, (**b**), offset and (**c**), technological sizes.

Generally, a standard full-profile ISO insert is used during the threading operation on a CNC lathe. The importance of the full-profile insert lies in the fact that its shape corresponds to h_3_ and R (Eqs. 1 and 2). Hence, when the total depth of cut is determined, the tool shape is fixed in relation to the thread pitch. The optimal position during the final pass of the full-profile insert can be seen in Fig. 5. Optimal operation occurs when the insert contour is centred in the tolerance zone, corresponding in the most precise region which is the pitch diameter.

Based on the thread tolerances, the total depth of cut can be calculated from the nominal diameter. In this new method, the nominal diameter is important because the threading command in the G-code is programmed from this diameter (e.g. M12 → X12). The shape and size of the insert depend on the pitch; therefore, the total depth of cut depends only on three parameters (Eq. 5):

5 $$\:{a}_{p}={h}_{3}+\frac{\left|es\right|}{2}+\frac{{T}_{d2}}{4}=0.6134\cdot\:p+\frac{\left|es\right|}{2}+\frac{{T}_{d2}}{4}$$

where |es| is the absolute value of the fundamental deviation according to ISO 965-1:2026^25^ Table 1, and (T_d2_) represents the pitch diameter tolerance of the external thread, according to^25^.

The method can be implemented through the following steps:

1. Determine the thread size, pitch, and tolerances.
2. Select full-profile insert according to the pitch (P).
3. Check the nose radius, it should equal with R (Eq. 2).
4. Select the appropriate es and T_d2_ values from the standard^25^ Tables 1).
5. Calculate the total depth of cut (a_p_).
6. Create the appropriate G-code for CNC turning.

*Example (case study 1)*.

1. The technical drawing specifies M12, which corresponds to M12 × 1.75–6 g.
2. 16ER1.75ISO full profile insert is selected from SECO^26^ p. 129.
3. According to the catalogue, the nose radius (RE) is 0.25, which is practically the same as the value obtained from Eq. (2): $\:R=0.14434\cdot\:P=0.14434\cdot\:1.75\approx\:0.2526$
4. According to the standard $\:es=0.034\:mm$ and $\:{T}_{d2}=0.15\:mm$.
5. $$\:{a}_{p}=0.6134\cdot\:p+\frac{\left|es\right|}{2}+\frac{{T}_{d2}}{4}=0.6134\cdot\:p+\frac{0.034}{2}+\frac{0.15}{4}\approx\:1.128\:mm$$
6. For the Sinumerik 808D control system, the G-code is shown in Table 1. The nominal diameter and the corresponding total depth of cut are highlighted in bold.

**Table 1:**
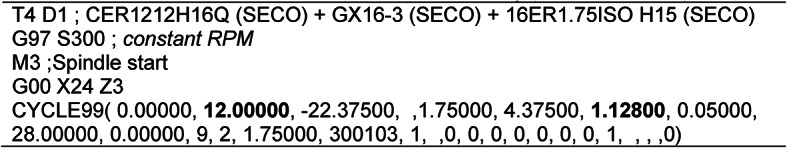
G-code that creates the correct thread at turning (Sinumerik 808D).

After the production the thread was checked with thread ring gauges (ISO 1502), because it was available in case of coarse pitch with default 6 g tolerances. The verification was successful at the first time.

## Method 2 for milling external thread

The main differences between thread turning and thread milling is related to the shape of the thread profile. Solid carbide cutters and milling cutters with inserts are available in a wide range of types. Some can be used only for internal or external threads, while others are suitable for both cases. Also, there are full profile and partial profile versions. Even full-profile tools generally do not conform to Eqs. (1) and (2). Moreover, in most cases the nose radius is not indicated in the catalogue. Due to these conditions, the previous calculation method cannot be used.

**Fig. 6:**
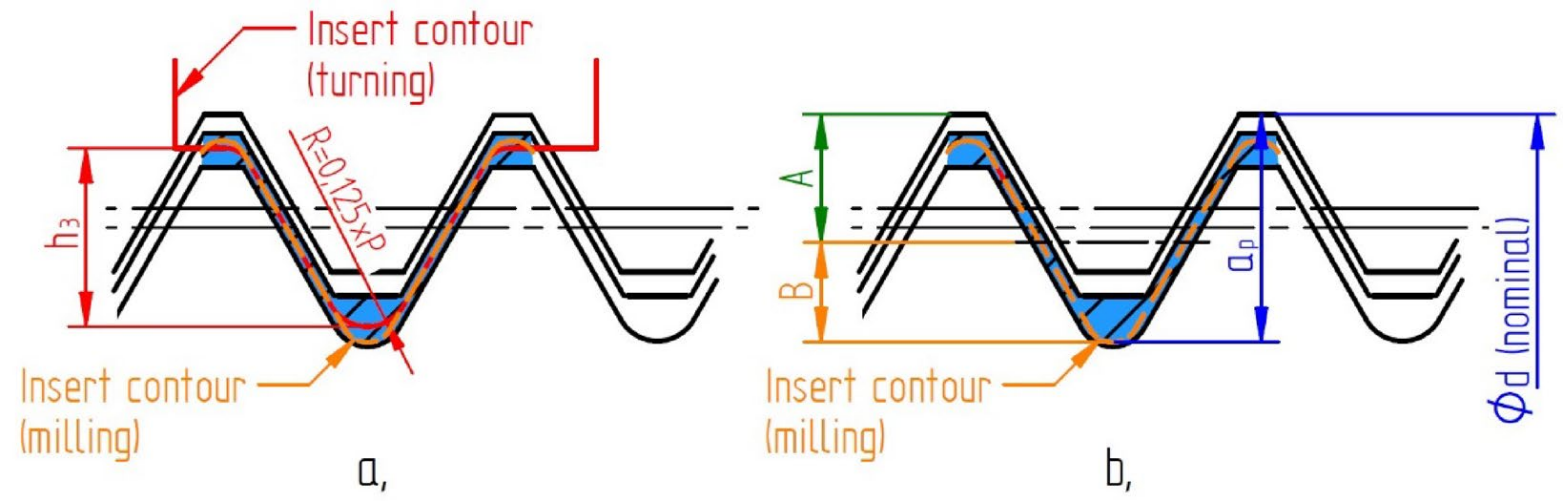
Differences between turning and milling profiles (**a**), and the key dimensions of the method (**b**).

**Fig. 7:**
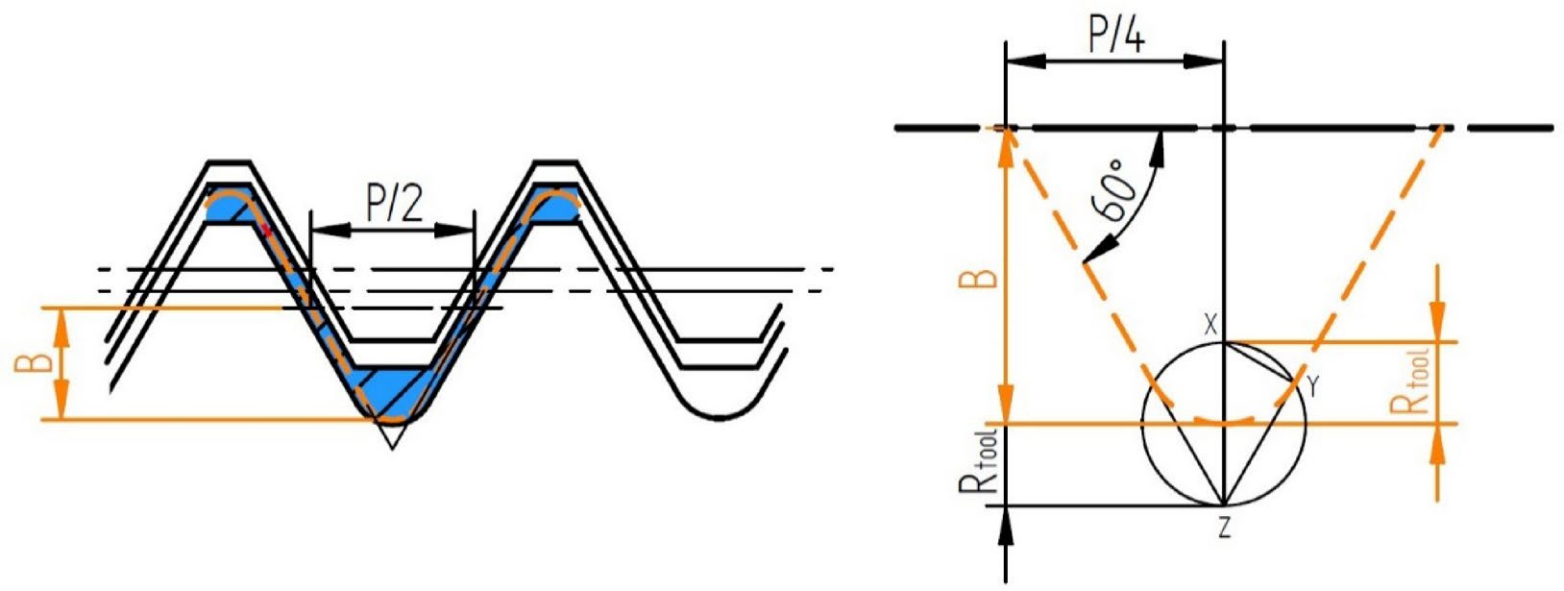
The B component, which depends on the insert profile.

This calculation is based on the fact that, the shape of the tool or insert profile is generally the same: a 60° angle with a fillet as the nose radius (Fig. 6a). If the radius is smaller, the tool must cut deeper, which is not a problem since the minor diameter has a larger tolerance zone. The radius should be included in the calculation, as it can be measured using an external tool setter, tool microscope, or projector (and in some cases it is specified in the catalogue). The total depth of cut can be calculated (Fig. 6b) based on the nominal and average pitch diameters (A, Eq. 6) and the insert profile (B, Fig. 7).

6 $$\:A=d-\overline{{d}_{2}}=d-\frac{{d}_{2\:min}+{d}_{2\:max}}{2}$$

The XYZ triangle is a right-angled triangle (a Thales circle is drawn on the figure). Moreover, the XZY angle is 30°, so point Z also belongs to the non-truncated triangle. Based on these facts, B can be calculated according to Eqs. (7) and (8).

7 $$\:tan(60^\circ\:)=\frac{B+{R}_{tool}}{P/4}$$

8 $$\:B=tan(60^\circ\:)\cdot\:\frac{P}{4}-{R}_{tool}$$

The total depth of cut in thread milling is given by Eq. (9). It depends only on the nominal diameter, the average pitch diameter, and the tool nose radius.

9 $$\:{a}_{p}=A+B=\:d-\frac{{d}_{2\:min}+{d}_{2\:max}}{2}+tan(60^\circ\:)\cdot\:\frac{P}{4}-{R}_{tool}$$

The method can be implemented through the following steps:

1. Determine the thread size, pitch, and tolerances.
2. Select an insert according to the pitch (P).
3. Determine the nose radius.
4. Calculate the d_2min_ and d_2max_ values according to the standard.
5. Calculate the total depth of cut (a_p_).
6. Create the appropriate G-code for CNC milling.

This new calculation method can also be used in the case of CNC thread turning when the nose radius does not conform to Eq. (2). As the function is the same as in the previous method, the benefits are identical (faster and cheaper production). On the other hand, the nose radius must be measured before starting programming.

*Example (case study 2)*.

1. The technical drawing specifies: M64 × 2–4 h.
2. CERATIZIT Modu thread milling insert (16M2.0) is selected.
3. The nose radius is 0.26 mm according to the external tool setter measurement.
4. Pitch diameters are 62.589 and 62.701 mm.
5. $$\:{a}_{p}=64-\frac{62.701+62.589}{2}+tan\left(60^\circ\:\right)\cdot\:\frac{2}{4}-0.26=1.2835\:mm$$

## The proper G-code was generated with EdgeCAM

Purchasing GO and NO-GO gauges was not worthwhile (mostly coarse pitch with 6 g tolerance was available without special order), because it was a single piece production. To confirm the product, a projector was used to check the external thread. With the measurement projector, the diameters and the shape can also be determined (similar to a profilometer). Based on the specific insert shape and the corresponding calculated depth of cut, the workpiece met the requirements on the first attempt. The confirmation could be done without additional measurement cost.

## Method 3 for milling and turning internal thread

The manufacturing of internal threads depends not only on the insert and thread tolerances but also on previous operations. Before the threading operation can be done, drilling, pocket milling (CNC milling), or boring (CNC turning) should be done.

**Fig. 8:**
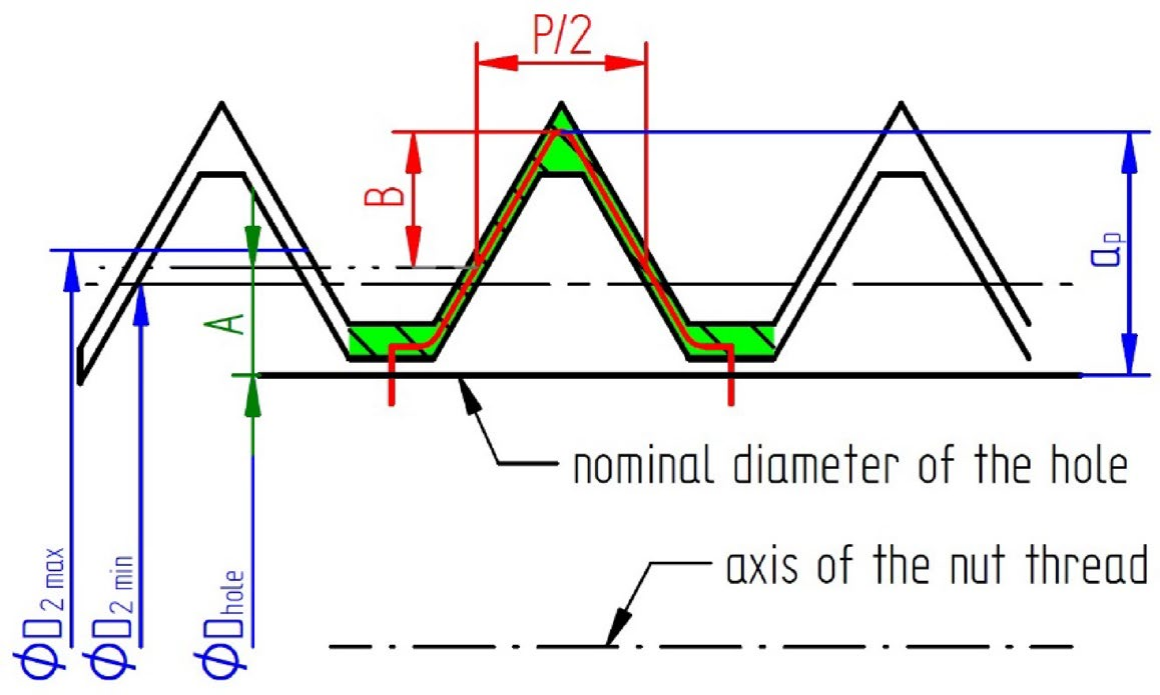
Relevant sizes for internal thread calculations.

To define the necessary depth of cut, a reference diameter (typically the nominal diameter of the pre-drilled hole) must be selected. Like Method 2, the nose radius of the tool should also be determined, because there are no standardized requirements related to the radius. Figure 8 shows the typical sizes for the calculation. It is clearly seen that the nominal diameter only determines the minimum size, and only the shape controls the maximum value, which is automatically achievable with the proper insert profile. Equation (9) should be slightly modified to calculate the total depth of cut in the case of an internal thread (Eq. 10):

10 $$\:{a}_{p}=A+B=\frac{{d}_{2\:min}+{d}_{2\:max}}{2}-{D}_{hole}+tan(60^\circ\:)\cdot\:\frac{P}{4}-{R}_{tool}$$

The method can be implemented through the following steps:

1. Determine the thread size, pitch, and tolerances.
2. Select an insert according to the pitch (P).
3. Determine the nose radius.
4. Calculate the d_2min_ and d_2max_ values according to the standard.
5. Define the reference diameter, e.g. the nominal size of the drilled hole.
6. Calculate the total depth of cut (a_p_).
7. Create the appropriate G-code for CNC milling or CNC turning.

*Example (case study 3)*.

1. The technical drawing specifies M20 × 1.5, with default tolerances 6 H.
2. Fraisa Multi-range thread milling cutter P1.5 (d_min_=18 mm) is selected.
3. The nose radius is 0.14 mm, measured with an external tool setter.
4. The pitch diameters are 19.026 and 19.216 mm.
5. The pocket-milled hole diameter is 18.4 mm.
6. $$\:{a}_{p}=\frac{19.026+19.216}{2}-18.4+tan\left(60^\circ\:\right)\cdot\:\frac{1.5}{4}-0.14=0.87\:mm$$
7. The proper G-code was generated with EdgeCAM

Since this is a fine thread, a thread plug gauge was not available in the workshop. However, this thread type is widely used in the customer’s applications; therefore, they maintain custom gauges for inspection. Consequently, the verification of the workpiece was carried out by the customer. The workpiece was accepted on the first attempt.

## Usability and limitations of the methods

**Fig. 9:**
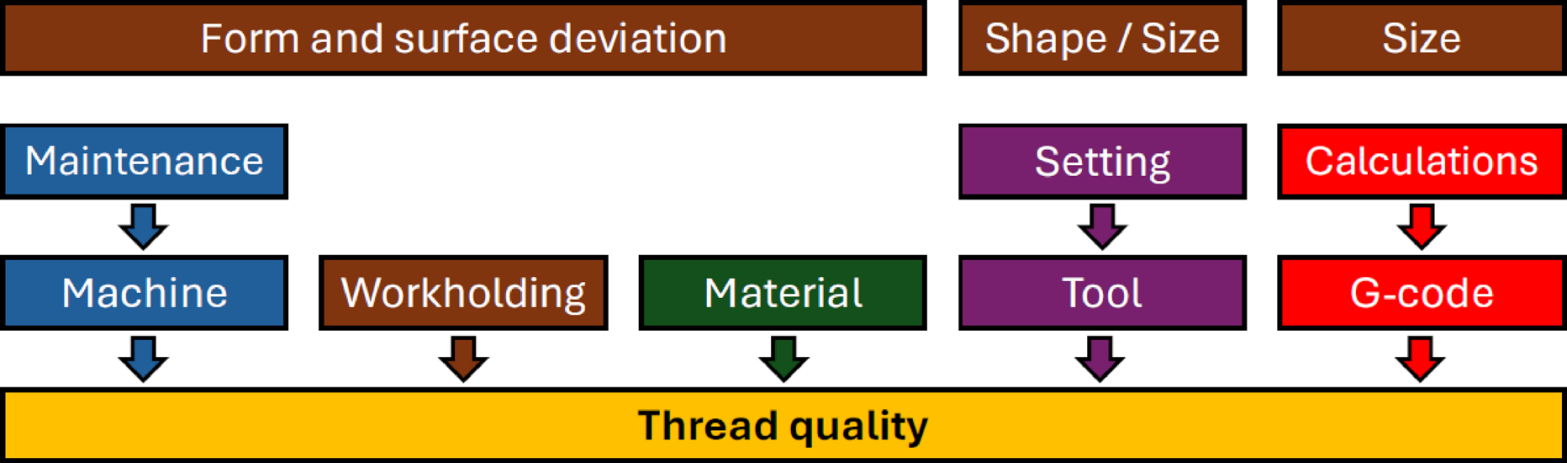
Connection between thread quality and the most important manufacturing factors.

In thread manufacturing, many factors influence thread quality (Fig. 9). The proposed method addresses the fact that, in most cases, the total depth of cut defined in the G-code is not accurate. Based on the theoretically exact geometry, this source of error can be reduced. The current industrial practice relies on GO and NO-GO gauges, with which the operator can identify nonconformities and apply wear compensation to adjust the tool path. After several iterations, the CNC machine is correctly set, and production can proceed. This process only influences size deviation, which can result from an incorrect total depth of cut and tool positioning. Other errors, such as shape, form, and surface deviations, can be controlled only to a limited extent using gauges. These errors require alternative measurement methods, such as profile scanning and surface roughness measurement.

The new methods help achieve the correct size, but their limitations are clear. They do not eliminate other types of errors; moreover, they address only one factor (albeit an important one) contributing to dimensional deviation. On the other hand, this is practically the only error that can be measured using gauges inside the CNC machine, and it therefore becomes the primary parameter that can be adjusted during CNC setup.

To achieve conformity in the first case, several additional requirements must be fulfilled. Tool setting plays a critical role, as it directly influences the size as well. If the tool overhang is accurately determined (e.g., using a tool setter) prior to machining, the tool will be positioned at the optimal location, which eliminates another source of size deviation. In single-part production, the tool is dedicated to a specific threaded operation, so tool overhang is determined just before manufacturing. Moreover, tool wear is negligible; therefore, the tool overhang does not change significantly. In this type of production, insert geometry is also verified before machining, ensuring that the original tool shape is confirmed, which is practically unaffected due to the low number of produced parts (typically 1–10 pieces, with negligible wear).

Cutting forces may also influence thread quality through workpiece deflection (form deviation). To mitigate this effect, an additional finishing pass without radial depth of cut can be applied, reducing the final chip cross-section and cutting forces, this resulting less deformation as well. Moreover, if possible, the use of alternative raw materials (e.g., heat-treated materials) or improved workholding can also help to mitigate this effect.

Unfortunately, certain critical effects cannot be fully avoided, such as vibration, material inhomogeneity, and tool fracture. However, during and after machining, several indicators (e.g., sound, surface quality, burr formation, and post-process tool condition) can be used to identify non-ideal process conditions, providing additional information about the machining process. Regular machine maintenance can also help reduce these influencing factors.

**Fig. 10:**
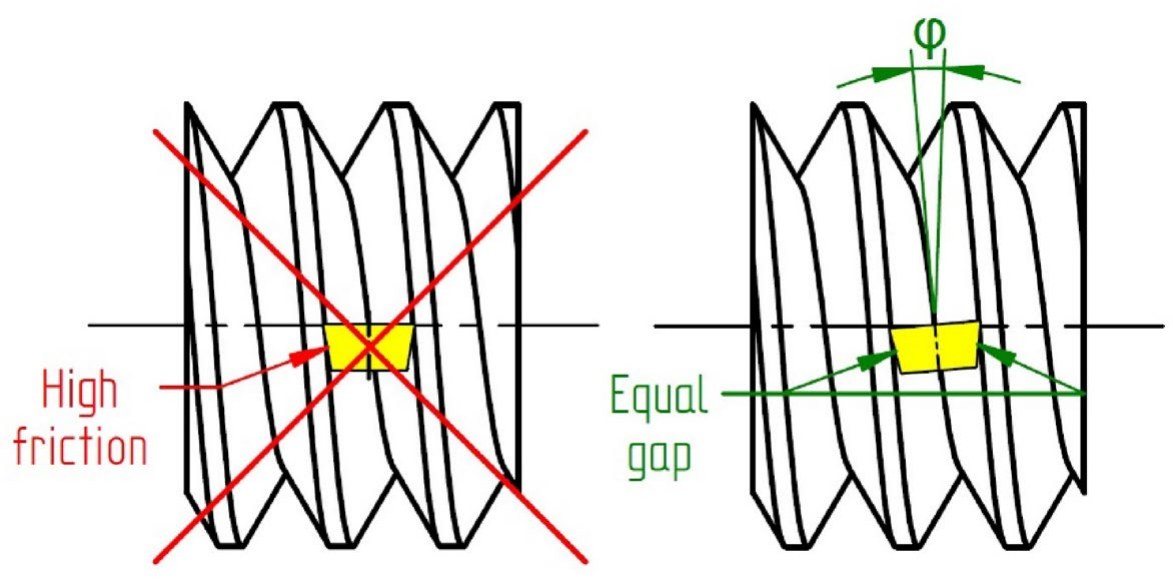
Effect of shim selection on insert orientation and threading performance.

Other theoretical limitations also influence thread quality. For example, proper shim selection is required for optimal threading operations (Fig. 10^26^) . Another example is the partial profile insert. In this case, the thread insert is only responsible for forming the root and the flanks of the thread, but not the crest. The crest is machined in a previous straight turning operation. Based on the fact that the nose radius of the partial profile insert never matches the standard profile, deviations may occur. Additionally, tool setting of the threading tool and proper calculations are not sufficient to fully compensate for dimensional deviations, because the previous machining operation also has an influence. In this case, additional measurement is necessary. It must be highlighted that the second and third methods can be applied to partial-profile inserts without any modification. Although the total depth of cut may be correct and the threaded connection can be assembled, the resulting thread profile does not fully comply with the standards. Based on this, the preferred choice should always be a full-profile insert, as also stated in the manufacturers’ catalogues.

Overall, if tool selection, tool setting and machining conditions are properly controlled, with a good G-code can help to the first manufactured part can be accepted.

In the case of series production some of the above-mentioned errors may significantly affect manufacturing performance. To reduce costs, cutting inserts are often used for extended periods compared to single-piece production; consequently, significant tool wear may occur. This wear changes the tool overhang and directly affects thread accuracy. It should be noted that the thread shape does not depend solely on the nose radius, but also on the two flanks of the insert, whose orientation is defined by the 60° profile angle. According to manufacturer recommendations, optimal chip formation is typically achieved using modified flank infeed. Therefore, not only the nose radius but also both side edges of the insert are involved in chip formation. Insert wear primarily modifies the root radius, which is generally less critical due to the larger root diameter tolerance zone. On the other hand, uniform wear on both flanks directly influences thread size. In practice, significant wear usually occurs at the nose of the insert before flank wear becomes critical.

The operator should use GO and NO-GO gauges to verify the thread and apply the necessary wear compensation in the controller. In addition, the use of an extra finishing cut (to minimize the risk of workpiece displacement) may be applied to avoid the formation of nonconforming threads; however, this approach increases machining cycle time and is therefore not practical in mass production.

Operators are often not highly skilled and may not always be present at the machine during threading operations. As a result, they may not recognize secondary indicators of suboptimal cutting conditions.

Although in mass production, accurate calculation of the total depth of cut can accelerate first-part confirmation and reduce the need for tool-change adjustments, as only wear compensation needs to be applied.

## Conclusion

Competition between companies drives continuous improvement. In the case of single-piece or small-batch production, a high ratio of waste can occur if the first product does not meet the requirements. Previously, such waste was acceptable, but nowadays it is no longer tolerated due to cost and time constraints, as well as the unnecessary environmental burden. This industrial demand supports the present research aimed at establishing improved best practices.

Once a workshop reaches a higher technical level, it can change the manufacturing principles. With accurate CNC machines and proper tool setters, the threading operation can also be changed. To avoid costly, gauge-based machine settings, prior calculations can be used to define the geometrically proper depth of cut for threading.

Three methods were developed to address practical threading scenarios for both CNC turning and milling. Table 2 contains the main parameters.

**Table 2:** Comparison of the different calculation methods.

Method	Tool	Parameters	Risks
External thread – CNC turning	Standard height and radius	Size, pitch and thread tolerance	The insert is not standard
External thread – CNC milling	Fixed shape, free radius	Size, pitch and thread tolerance; nose radius	Measure the nose radius
Internal thread – CNC turning and milling	Fixed shape, free radius	Thread tolerance; nose radius; reference diameter	Measure the nose radius

Based on these calculations, the dimensionally correct G-code can be generated, which contributes to achieving waste-free production. The implementation of the methods in production was successful and speed up CNC machine setup processes based on the mention case studies. Moreover, for atypical thread sizes and tolerance classes, both measurement cost and lead time can be eliminated since gauge purchasing is not required.

Unfortunately, the practical implementation in single-piece production does not allow the creation of statistical evidence for first-part conformity; however, the above-mentioned examples demonstrate the feasibility of the concept. Moreover, it is clear that proper input parameters (tolerance-based total depth of cut) improve manufacturing performance and eliminate one practical limitation. On the other hand, limitations to achieving first-part conformity still remain. These mainly arise from form and shape deviations, as well as poor surface roughness. Without proper tool setup, which also influences size deviation, geometrically accurate G-code alone cannot ensure the required size tolerances.

## Supplementary Information

Below is the link to the electronic supplementary material.

Supplementary Material 1

## Data Availability

Availability of dataData available on request from the author.
