# Supplementary figures and images for: Novel calculation methods for geometrically accurate thread depth
Source: Sci Rep. 2026 May 18;16:21004. doi: 10.1038/s41598-026-53095-1 (PMC13342070; doi:10.1038/s41598-026-53095-1)

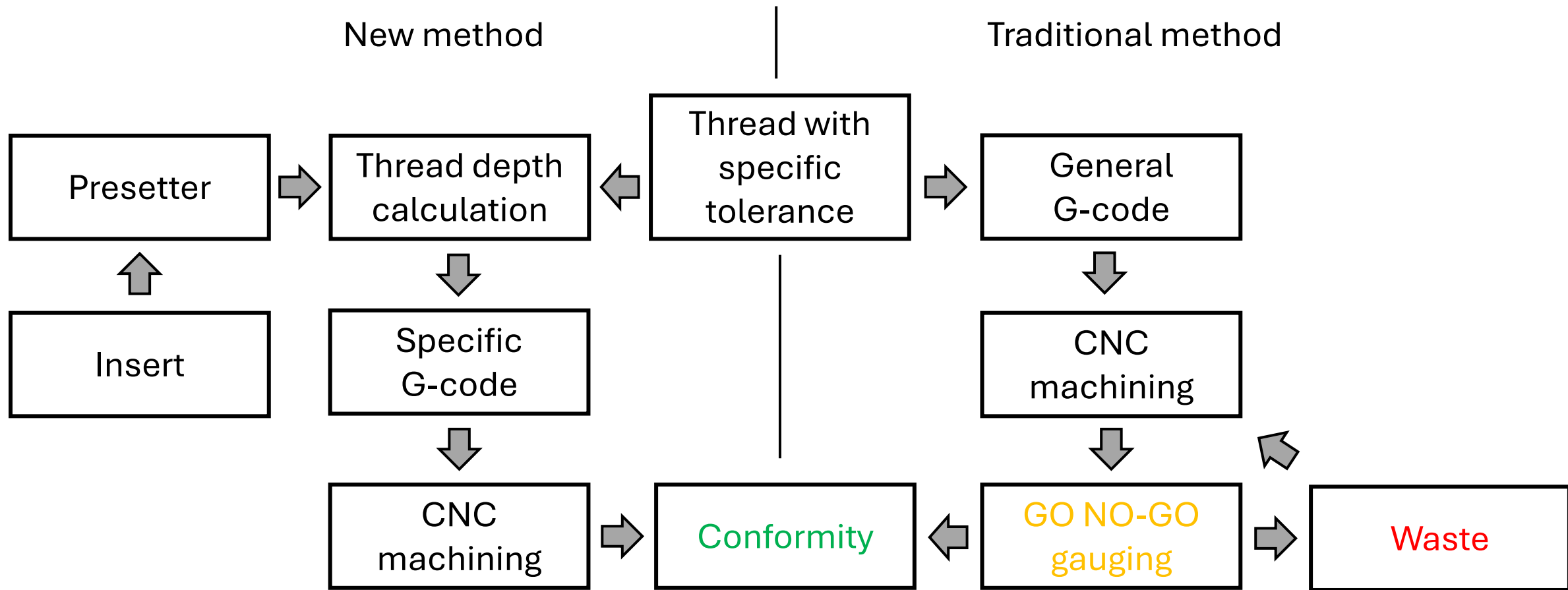

Supplement: Supplementary file 1 — Supplementary Material 1 [file 41598_2026_53095_MOESM1_ESM.pdf]
